# Deciphering the Role of Histone Modifications in Uterine Leiomyoma: Acetylation of H3K27 Regulates the Expression of Genes Involved in Proliferation, Cell Signaling, Cell Transport, Angiogenesis and Extracellular Matrix Formation

**DOI:** 10.3390/biomedicines10061279

**Published:** 2022-05-30

**Authors:** María Cristina Carbajo-García, Lucia de Miguel-Gómez, Elena Juárez-Barber, Alexandra Trelis, Javier Monleón, Antonio Pellicer, James M. Flanagan, Hortensia Ferrero

**Affiliations:** 1 Fundación IVI, IIS La Fe, 46026 Valencia, Spain; mcristina.carbajo@ivirma.com (M.C.C.-G.); lucia.demiguel@ivirma.com (L.d.M.-G.); elena.juarez@ivirma.com (E.J.-B.); apellicer@ivirma.com (A.P.); 2 Departamento de Pediatría, Obstetricia y Ginecología, Universidad de Valencia, 46010 Valencia, Spain; 3 Department of Surgery and Cancer, Imperial College London, London SW7 2AZ, UK; j.flanagan@imperial.ac.uk; 4 Hospital La Fe, 46009 Valencia, Spain; alexandratrelis@gmail.com (A.T.); monlesancho@gmail.com (J.M.); 5 IVIRMA Rome, 00197 Rome, Italy

**Keywords:** histone modification, gene expression, angiogenesis, extracellular matrix, uterine leiomyoma

## Abstract

Uterine leiomyoma (UL) is a benign tumor arising from myometrium (MM) with a high prevalence and unclear pathology. Histone modifications are altered in tumors, particularly via histone acetylation which is correlated with gene activation. To identify if the acetylation of H3K27 is involved in UL pathogenesis and if its reversion may be a therapeutic option, we performed a prospective study integrating RNA-seq (*n* = 48) and CHIP-seq for H3K27ac (*n* = 19) in UL vs MM tissue, together with qRT-PCR of SAHA-treated UL cells (*n* = 10). CHIP-seq showed lower levels of H3K27ac in UL versus MM (*p*-value < 2.2 × 10^−16^). From 922 DEGs found in UL vs. MM (FDR < 0.01), 482 presented H3K27ac. A differential acetylation (FDR < 0.05) was discovered in 82 of these genes (29 hyperacetylated/upregulated, 53 hypoacetylated/downregulated). Hyperacetylation/upregulation of oncogenes (*NDP,HOXA13,COL24A1,IGFL3*) and hypoacetylation/downregulation of tumor suppressor genes (*CD40,GIMAP8,IL15,GPX3,DPT*) altered the immune system, the metabolism, TGFβ3 and the Wnt/β-catenin pathway. Functional enrichment analysis revealed deregulation of proliferation, cell signaling, transport, angiogenesis and extracellular matrix. Inhibition of histone deacetylases by SAHA increased expression of hypoacetylated/downregulated genes in UL cells (*p* < 0.05). Conclusively, H3K27ac regulates genes involved in UL onset and maintenance. Histone deacetylation reversion upregulates the expression of tumor suppressor genes in UL cells, suggesting targeting histone modifications as a therapeutic approach for UL.

## 1. Introduction

Uterine leiomyomas (ULs) are monoclonal benign tumors arising from the myometrium (MM) that affect up to 25–30% of women of reproductive age [1,2]. Around 30% of these patients present symptoms, such as excessive uterine bleeding, infertility, or recurrent abortion [3]. Although the gold standard treatment is surgery, less invasive hormonal treatments are sometimes used to treat leiomyomas [4,5]. However, these treatments cause side effects such as menopausal symptoms or hepatic damage [6], and, once treatment is stopped, leiomyomas enlarge again [7]. For this reason, no effective therapy with minimal side effects is currently available to treat UL. The lack of efficient treatment could be because available medical options focus on the relief of symptoms and not in mechanisms implicated in UL development [8]. Therefore, identification of molecular mechanisms involved in UL pathogenesis could allow the development of new and more efficient treatments.

Although UL pathogenesis remains incompletely understood, many factors contribute to its development, including steroid hormones, growth factors and genetics. ULs develop after menarche and regress after menopause [9,10]. Their growth is affected by the concentrations of steroid hormones, especially estrogen and progesterone. Estrogen and progesterone act on the tissue’s mature cells and, through them, send paracrine factors to the stem cell population inducing its proliferation [11]. Therefore, UL and MM growth is dependent on these hormones. For this reason, hormonal treatments based on the inhibition of estrogen and progesterone production, such as gonadotropin releasing-hormone agonist (aGnRH), have been used for UL treatment [5]. In addition, genetic mutations have been described as a possible cause of UL development. In this regard, there are four established molecular subtypes of UL per mutations on different genetic drivers: MED12 mutations (70–75% of patients with UL [12]), HMGA2 rearrangements (20% of UL patients [13]), biallelic inactivation of FH (10.5% of UL patients [14]), and deletions affecting COL4A5 and COL4A6 (4% of UL patients [13]). However, factors such as race, diet, age, body mass index, and parity are also risk factors for UL [15], suggesting potential involvement of epigenetic mechanisms in UL development. Epigenetics include variations in the gene expression profile not caused by changes in the DNA sequence, resulting from processes such as DNA methylation, histone modification, and non-coding RNAs [16]. Genome-wide DNA methylation studies have revealed subsets of suppressed or overexpressed genes accompanied by aberrant promoter methylation [17,18,19], also evaluating DNA methylation in UL focusing on stem cell population [20] or mutation status [21]. Furthermore, differential promoter access resulting from altered 3D chromatin structure and histone modifications plays a role in regulating transcription of key genes thought to be involved in leiomyoma etiology [22]. These modifications are inherited somatically and are dynamic and reversible, which make them potential therapeutic targets.

Modification of histone proteins is a key epigenetic mechanism implied in the regulation of gene expression. These modifications occur at the N-terminal tail or the globular domains of core histones [23]. Epigenetic modifications of histone tails include acetylation, methylation, phosphorylation, ubiquitination, and SUMOylation. Histone acetylation is correlated with gene activation, whereas loss of acetylation (deacetylation) represses gene expression [24]. The enzymes participating in the addition of an acetyl group to histones are histone acetyltransferases (HATs), while histone deacetylases (HDACs) remove these marks [24,25]. HDACs are involved in the development of different tumors such as ovarian and breast cancer [26,27,28] and UL [9,23,29]. Specifically, HDAC activity was found to be higher in UL than in adjacent MM, suggesting that the transcription of genes implicated in the normal function of MM may be repressed due to a decrease in histone acetylation [9,29,30]. In addition, we previously described that inhibition of HDACs by suberoylanilide hydroxamic acid (SAHA) inhibits cell proliferation, cell cycle, extracellular matrix (ECM) formation and TGF-β3 signaling in human uterine leiomyoma primary (HULP) cells, suggesting that HDAC inhibitors may present a viable therapeutic option [29]. Aberrant status of acetylated Lysine 27 of histone 3 (H3K27ac) profile is implicated in several tumors such gastric, lung and ovarian cancers [31,32,33]. Since HAT/HDACs are dysregulated in UL, a holistic analysis of the interaction between gene expression and H3K27ac profiles in UL compared to MM could provide insight into key pathways and driver genes involved in UL pathogenesis that are under this histone modification control. Based on this, we aimed to further study the role of histone acetylation in UL and to identify if the histone mark H3K27ac is involved in UL development by integration of RNA-seq and CHIP-seq data. With this study, we describe the functional implications of an aberrant profile of the histone mark H3K27ac over gene expression in UL compared to adjacent MM.

## 2. Materials and Methods

### 2.1. Data/Samples Acquisition

RNA-seq data (GSE192354 and GSE142332) and the CHIP-seq data for the histone modification H3K27ac (GSE142332) were downloaded from the Gene Expression Omnibus (GEO, https://www.ncbi.nlm.nih.gov/geo/ (accessed on 10 September 2021)) of the National Center for Biotechnology Information (NCBI). In total, gene expression data of UL and adjacent MM of 31 Caucasian women (aged 31–48) was obtained from GSE192354, while RNA-seq and CHIP-seq H3K27ac data of UL and adjacent MM of 21 Caucasian/African American and Latin women (aged 41–52) were acquired from GSE142332. UL and MM tissue was obtained from women undergoing myomectomy or hysterectomy due to UL, and the origin of these tissues was confirmed through hematoxylin/eosin staining by examination of a pathologist.

### 2.2. CHIP-Seq Analysis

For CHIP-seq analysis of H3K27ac, the following bioinformatics analysis was performed within the R/Bioconductor (version 4.1.1) computing environment. A biomaRt package was used to bring in gene annotation data from Ensembl to R. With the data loaded into the workspace, peaks that were within the ±2 kb region from the transcription start sites (TSS) of a known human gene were defined as genes that present this modification. After centering and scaling fold-enrichment of signal values corresponding to peaks provided by CHIP-seq, Principal Component Analysis (PCA) and heatmap were performed and boxplot of H3K27ac histone mark status in UL and MM was represented with ggplot2 package. A Wilcoxon test (*p* < 0.05) was performed to test differences between H3K27ac status in UL vs. MM. Two samples were filtered out after quality analysis because of a low sequence depth.

### 2.3. RNA-Seq Analysis

Separate analyses were carried out for each dataset (GSE192354 and GSE142332). Raw count matrix derived from RNA-seq data libraries from GSE192354 was processed and subjected to statistical analysis within the R/Bioconductor (version 4.1.1) computing environment. PCA was performed to check concordance of DNA libraries. Differentially expressed genes (DEGs) were analysed using three different packages: DESeq2, edgeR and limma. RNA-seq data libraries from GSE142332 were analysed as previously described [34]. DEGs were obtained using DESeq2. Common DEGs between both datasets with an FDR-adjusted *p*-value < 0.01 and log2FC > 1 or <−1 were considered for the consequent analysis.

### 2.4. Correlation of H3K27ac and Gene Expression

Common DEGs resulting from RNA-seq analysis were integrated in each CHIP-seq data by selecting those for which a ChIP-seq peak was detected in the regulator region (TSS ± 2kb). A boxplot of the H3K27ac status for downregulated and upregulated genes in each group (UL and MM) was represented with a ggplot2 package. A Wilcoxon test (*p* < 0.05) was performed to test differences between H3K27ac status in the different groups. Differential peak enrichment analysis was performed using a linear model with a limma method. Peaks that were within the ±2 kb region from the TSS of the DEGs with FDR-adjusted *p*-value < 0.01 after limma analysis were defined as the significant differential modifications. A Venn diagram was used to identify hypoacetylated/downregulated and hyperacetylated/upregulated after H3K27ac analysis.

### 2.5. Functional Enrichment Analysis

Gene ontology (GO) analysis was conducted on selected genes which were hypoacetylated/downregulated and hyperacetylated/upregulated after H3K27ac analysis via Shiny Go (version 0.741) [35]. Biological processes and cellular components were considered to be statistically significant with FDR < 0.05.

### 2.6. Sample Collection

For gene expression validation and in vitro culture, human UL and adjacent MM (*n* = 10) tissue were collected from Caucasian premenopausal women aged 31–48 years without any previous hormonal treatment for the last three months and who were undergoing myomectomy or hysterectomy due to symptomatic UL at Hospital Universitario y Politécnico La Fe (Spain). This study was approved by the Clinical Ethics Committee at Hospital Universitario y Politécnico La Fe (Spain) (2018/0097), and all participants provided informed consent.

### 2.7. Validation of Gene Expression: qRT-PCR

Gene expression of selected DEG was validated in a distinct cohort of UL and adjacent MM (*n* = 10) by quantitative real-time PCR (qRT-PCR). Total RNA was extracted from tumor (UL) and normal tissues (MM) with TRIzol reagent (Fisher Scientific, Waltham, MA, USA), and complementary cDNA was synthesized employing a PrimeScript RT reagent kit (Takara, Kusatsu, Japan). Expression of genes *NDP, HOXA13, COL24A1, IGFL3, CD40, GIMAP8, IL15, GPX3* and *DPT*, was analysed by qRT-PCR with a StepOnePlus system (Applied Biosystems, Waltham, MA, USA) and PowerUp Sybr Green (ThermoFisher Scientific, Waltham, MA, USA). GAPDH gene was employed as housekeeping for gene expression normalisation. The ΔΔCt method was used to calculate fold change. Primers were designed using Primer Quest Tool (Integrated DNA Technologies, Coralville, IA, USA).

### 2.8. SAHA Treatment and Gene Expression Analysis in Human Uterine Leiomyoma Primary Cells

To evaluate the effect of SAHA (Abcam, Cambridge, UK) on the selected downregulated and hypoacetylated genes, HULP cells were isolated from UL tissues (*n* = 10) from selected women, as previously described [36] and treated with 0 μM (0.01% DMSO as a control) or 10 μM of SAHA for 48h. Then, total RNA was extracted from HULP cells using a Qiagen RNeasy Mini kit, and cDNA was synthesized using a Takara PrimeScript RT reagent kit; qRT-PCR was performed to evaluate gene expression *CD40*, *GIMAP8*, *IL15, GPX3* and *DPT* in HULP cells treated with or without SAHA, as described above.

### 2.9. Statistical Analysis

Omics data analysis was performed using R (version 4.1.1). Graphics were created using the R core package and packages gplots, ggplot2, as well as GraphPad Prism 8.0. Gene expression validation analysis was conducted with GraphPad Prism 8.0 employing Student’s t-test or Wilcoxon test; *p* < 0.05 was considered statistically significant.

## 3. Results

### 3.1. Global H3K27ac CHIP-Seq Peak Profile in Uterine Leiomyoma Tissue Compared to Adjacent Myometrium

**Figure 1:**
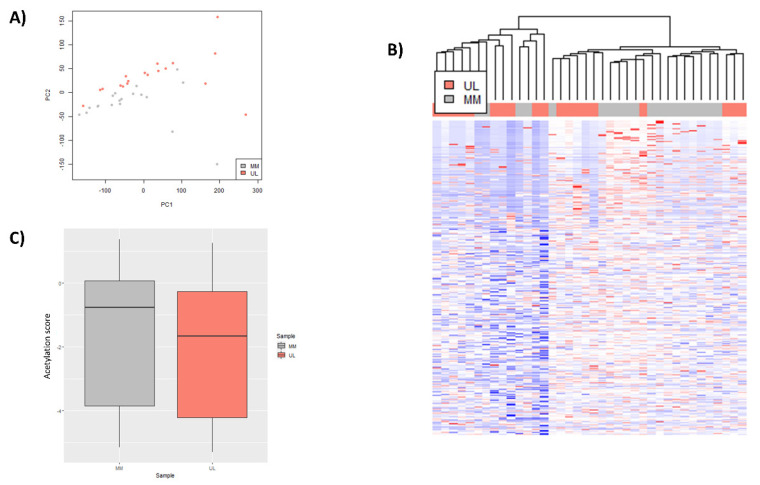
Global acetylation status of H3K27 in uterine leiomyoma compared to adjacent myometrium tissues from GSE142332: (**A**) principal component analysis (PCA) of global H3K27ac profile in uterine leiomyoma (UL) (pink) and adjacent myometrium (MM) (blue) (*n* = 19/group); (**B**) heatmap based on fold-enrichment score of genes with a CHIP-seq H3K27ac peak in TSS ±2000 bp after unsupervised clustering of UL (pink) and MM (gray) (*n* = 19/group); color scale ranges from red for higher normalized fold-enrichment score to blue for lower levels; (**C**) boxplot representing distribution of normalized fold-enrichment score for each peak in UL (pink) compared to adjacent MM (gray) samples (*n* = 19/group), representing global H3K27ac status (*p*−value < 2.2 × 10^−16^).

To determine the general H3K27 acetylation profile in human UL compared to adjacent MM tissue, an exploratory analysis of all peak signal values was performed. Principal component analysis (PCA) revealed a separation between UL and adjacent MM (Figure 1A). Clustering analysis showed common patterns in UL and MM, tending to form groups, as observed in a heatmap (Figure 1B). A boxplot of genes whose regulator region presented H3K27ac after CHIP-seq showed a lower amount of global H3K27ac peak enrichment level in UL compared to MM (*p*-value < 2.2 × 10^−16^), suggesting a global hypoacetylation of UL (Figure 1C).

### 3.2. Selection of Relevant Differentially Expressed Genes

**Figure 2:**
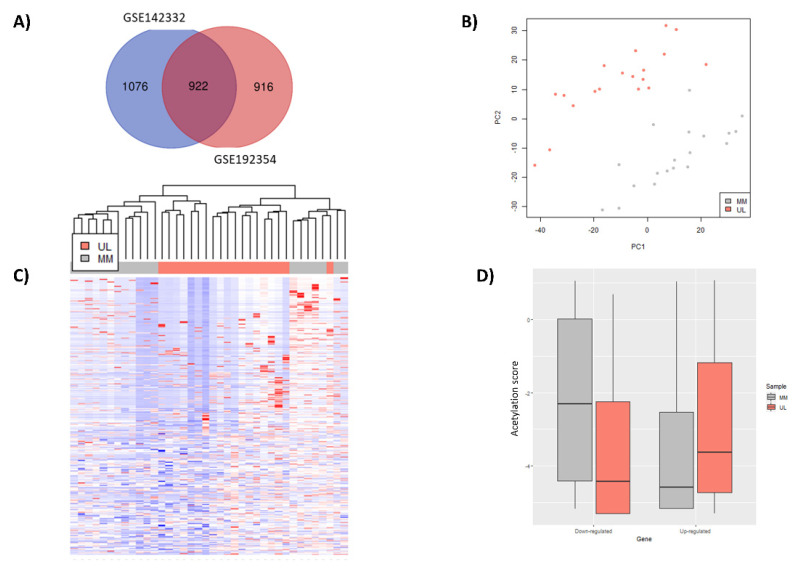
Identification of selected differentially expressed genes and description of their H3K27ac status in UL compared to MM tissue: (**A**) Venn diagram representing common DEGs (FDR-adjusted *p*-value < 0.01, log2FC > 1 or <−1) between GSE192354 (*n* = 28) and GSE142332 (*n* = 19) samples; (**B**) PCA of global H3K27ac profile and (**C**) Heatmap based on fold-enrichment score of 82 selected DEGs in common in both GSE192354 and GSE142332 whose promoter region presented a peak after CHIP-seq after unsupervised clustering of uterine leiomyoma (UL) (pink) and adjacent myometrium (MM) (gray) (*n* = 19/group); color scale ranges from red for higher normalized fold-enrichment score to blue for lower levels; (**D**) boxplot representing distribution of normalized fold-enrichment score for each peak of downregulated and upregulated genes in UL (pink) compared to adjacent MM (gray) samples (*n* = 19/group), representing H3K27ac status in each group of genes (*p*-value < 2.2 × 10^−16^).

To identify relevant differentially expressed genes involved in UL development, we integrated gene expression data obtained from two different studies. First, count matrix of RNA obtained from GSE192354 was analysed using the three most widely used packages for differential expression analysis: limma, DESeq2 and edgeR. The selection of overlapping differentially expressed genes resulting from these three analyses showed 1837 DEGs significant (FDR-adjusted *p*-value < 0.01) and with a high difference of expression (log2FC > 1 or < −1) in UL compared to MM (Figure 2A), with 1175 upregulated and 662 downregulated. Similarly, 1998 DEGs with an FDR-adjusted *p*-value < 0.01, log2FC > 1 or < −1 were obtained from GSE142332 after DESeq2 analysis, with 1106 upregulated and 892 downregulated. After intersection of both outcomes, 922 genes were revealed as common DEGs in UL compared to MM samples from both datasets (Figure 2A), with 559 upregulated and 363 downregulated. These genes were considered as relevant DEGs in UL for further analysis.

### 3.3. Identification Differentially Expressed Genes with an Aberrant H3K27ac Mark in Uterine Leiomyoma Tissue Compared to Adjacent Myometrium

Next, we aimed to evaluate those genes whose change of expression was associated with a differential H3K27ac status. Among the 922 genes selected for this analysis after RNA-seq, 482 (52.3%) presented the histone mark H3K27ac around the TSS ± 2 kb. A PCA of CHIP-seq data of these genes showed a clear separation of tumor (UL) and control (MM) samples (Figure 2B), indicating a different behaviour of H3K27ac profile of relevant selected genes in UL compared to adjacent MM, as confirmed by heatmap (Figure 2C). Additionally, a boxplot of fold-enrichment score of H3K27ac peaks representing downregulated and upregulated genes demonstrated that downregulated genes presented a lower fold-enrichment score of H3K27ac peaks (*p*-value < 2.2 × 10^−16^) in UL versus MM, while upregulated genes exhibited a higher fold-enrichment score (*p*-value < 2.2 × 10^−16^) in UL versus MM (Figure 2D). Differential peak enrichment analysis showed that 82 DEGs presented differential acetylation (FDR < 0.05) in UL compared to MM, with 29 hyperacetylated/upregulated and 53 hypoacetylated/downregulated (Supplemental Table S1).

### 3.4. Functional Implications of Differentially Expressed Genes Associated with Aberrant H3K27 Acetylation in Uterine Leiomyoma Tissue Compared to Adjacent Myometrium

**Figure 3:**
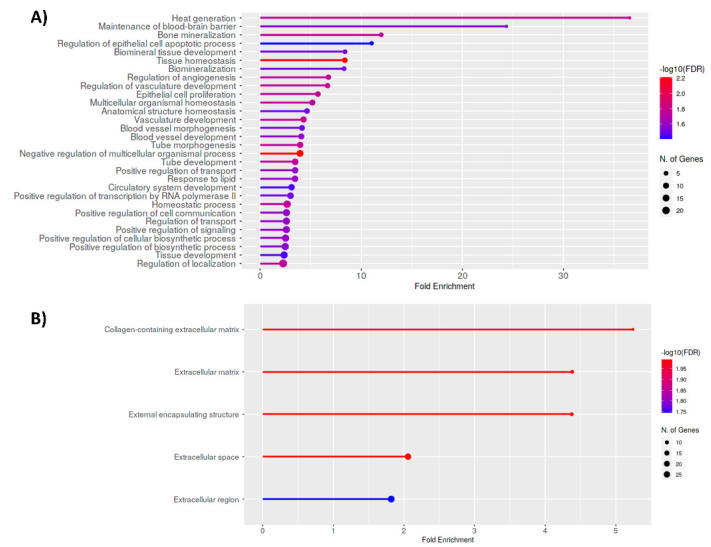
Functional enrichment analysis of differential expressed genes associated with aberrant H3K27ac status in UL vs. MM; most significant: (**A**) biological processes; and (**B**) cellular components obtained after functional enrichment analysis of all selected aberrantly acetylated DEGs in UL vs. MM tissues; FDR < 0.05.

Functional enrichment analysis of 82 DEGs associated with a different H3K27ac profile revealed 30 biological processes significantly deregulated in human UL versus MM that were mainly related to cell proliferation, cell signaling and cell transport and angiogenesis, key pathways in tumor pathogenesis (Figure 3A). In addition, cellular components were found to be significantly enriched in UL, which were all related to an alteration of the extracellular matrix, one of the key features of UL (Figure 3B).

### 3.5. Validation of Hypoacetylated/Downregulated and Hyperacetylated/Upregulated Genes

**Figure 4:**
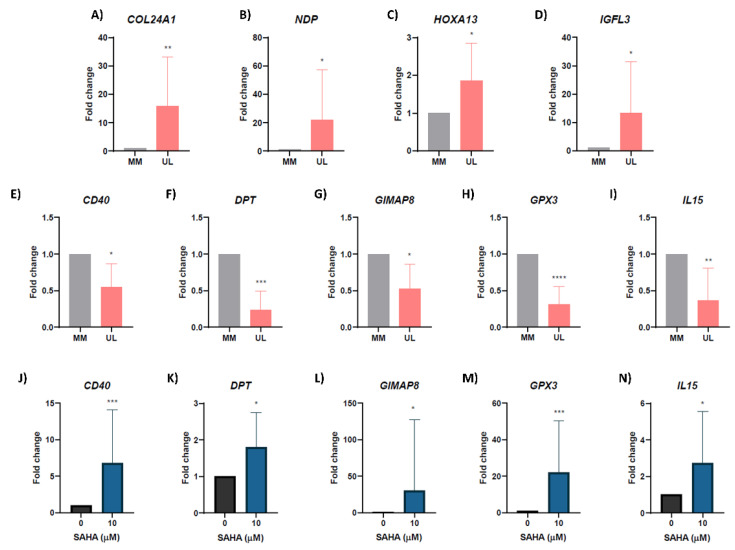
Validation of RNA-seq results and gene expression analysis in human uterine leiomyoma primary cells after SAHA treatment; expression levels of: (**A**) *COL24A1*; (**B**) *NDP;* (**C**) *HOXA13*; (**D**) *IGFL3*; (**E**) *CD40*; (**F**) *DPT*; (**G**) *GIMAP8*; (**H**) *GPX3;* and (**I**) *IL15* in the validation set of UL compared to adjacent MM (*n* = 10); gene expression levels of hypermethylated/downregulated genes: (**J**) *CD40*; (**K**) *DPT*; (**L**) *GIMAP8*; (**M**) *GPX;* and (**N**) *IL15* in human uterine fibroid primary (HULP) cells treated with 0 μM (control) or 10 μM of SAHA for 48 h (*n* = 10). Gene expression was analyzed by qRT-PCR, quantified by the ΔΔCt method and expressed as fold regulation. * *p* < 0.05; ** *p* < 0.01; *** *p* < 0.001; **** *p* < 0.0001.

To highlight the importance of the genes selected after integration of RNA-seq and CHIP-seq studies, gene expression of 10 genes which presented a differential H3K27ac status was validated in a different set of patients. These genes were selected as key genes of the enriched functions and based on their role in tumorogenesis after a bibliographic search among all hyperacetylated/upregulated and hypoacetylated/downregulated genes. The qRT-PCR corroborated the significant upregulation of *COL24A1* (fold-change = 15.83, *p* = 0.003), *NDP* (fold-change = 26.38, *p* = 0.037), *HOXA13* (fold-change = 1.86, *p* = 0.041) *and IGFL3* (fold-change = 13.26, *p* = 0.031) in a separate cohort of UL compared to adjacent MM (Figure 4A–D). Likewise, qRT-PCR confirmed the downregulation of *CD40* (fold-change = 0.54, *p* = 0.010), *DPT* (fold-change = 0.25, *p* = 0.002), *GIMAP8* (fold-change = 0.52, *p* = 0.015), *GPX3* (fold-change = 0.30, *p* < 0.0001) and *IL15* (fold-change = 0.36, *p* = 0.005) (Figure 4E–I).

### 3.6. Inhibiting Histone Deacetylases Reverses Expression of Hypoacetylated/Downregulated Genes in Human Uterine Leiomyoma Primary Cells In Vitro

To corroborate that histone acetylation of H3K27 is really affecting the expression of tumor suppressor genes in UL, we assessed the role of inhibition of HDACs on restoring the expression of genes controlled by H3K27ac, by inhibition of HDACs in human uterine leiomyoma primary (HULP) cells using SAHA treatment. Expression of the previously selected hypoacetylated and downregulated genes in UL was evaluated by qPCR after treatment with SAHA at 0 μM and 10 μM in HULPs. Results showed that inhibition of deacetylation by SAHA treatment significantly upregulated expression of tumor suppressor genes *CD40* (fold-change = 6.78, *p* = 0.001), *DPT* (fold-change = 1.80, *p* = 0.033), *GIMAP8* (fold-change = 30.67, *p* = 0.042), *GPX3* (fold-change = 22.15, *p* = 0.001) and *IL15* (fold-change = 2.71, *p* = 0.018) and in HULP cells (Figure 4J–N).

## 4. Discussion

Uterine leiomyomas are a major gynaecological disease with a great impact on women’s health, being a main cause of infertility. Despite the personal and economic consequences of this tumor, its pathology remains unclear. Recently, epigenetics has emerged as a new mechanism that may be involved in UL formation [23,30]. The epigenomic studies pertaining to UL pathogenesis have mainly focused on DNA methylation [20,21,37]. However, histone modifications also have the potential to play an important function in chromatin alterations, and therefore, it is necessary to fully explore the effect of histone acetylation on the expression of genes involved in the pathogenesis of this disease. In a previous study, we demonstrated that histone deacetylase inhibitors may present a viable therapeutic option for UL [29]. Herein, we further studied the role of histone acetylation over gene expression by identifying if the modification H3K27ac, which is altered in several tumors, is involved in UL pathogenesis and supports the importance as a new therapeutic approach to treat UL patients. Our results showed that H3K27ac regulates genes implicated in key processes of UL pathogenesis such as cell proliferation, cell signaling and cell transport, angiogenesis and ECM formation, and histone deacetylation reversion may represent a therapeutic approach to treat UL.

Post-translational modifications of histones hold importance in the epigenomic regulation of gene expression. Histone acetylation is correlated with gene expression, while deacetylation leads to repression of gene transcription [24]. After analysing the general H3K27 acetylation profile of promoter regions (TSS ± 2 kb) of human genes in UL compared to adjacent MM tissue, we found a different pattern of the histone mark H3K27ac. Specifically, a lower amount of global H3K27ac was observed in UL compared to MM, suggesting a global hypoacetylation of H3K27 in UL. The reduced H3K27 acetylation would lead to a decrease in the expression levels of genes that suppress tumor development. Accordingly, previous studies have shown that a global DNA hypermethylation is related to the downregulation of tumor suppressor genes involved in tumor development [17,21,34]. Although the role of histone modifications in UL is less understood compared to DNA methylation, recent publications have emphasized the significance of H3K27ac, H3K4me3 and H2A.Z in enhancers and promotors, finding differential features between UL subtypes based on the mutation status [22,34,38]. Herein, an altered pattern of H3K27ac in UL compared to MM regardless of their mutational subtype suggests its role in UL development and treatment.

The aberrant status of H3K27ac in UL can explain the altered chromatin structure, which aids in developing the UL-specific gene dysregulation resulting in its pathogenesis. Therefore, we selected key DEGs involved in UL development whose change of expression was associated with the histone mark H3K27ac. Among the 922 DEGs described by integration of RNA-seq analyses, 482 presented the histone mark H3K27ac around the promotor, and 82 of them exhibited a differential H3K27ac status in UL compared to MM, with 29 hyperacetylated/upregulated and 53 hypo-acetylated/downregulated.

To further analyse the new molecular targets involved in UL pathogenesis that are associated with H3K27ac, we reviewed the literature for the 82 DEGs regulated by H3K27ac and found that these genes present multiple functions, being potential key effecters of tumor development and maintenance. We found hyperacetylation/upregulation of oncogenes such as *NDP, HOXA13, COL24A1* and *IGFL3,* with *NDP* and *IGFL3* not previously related to UL. *NDP* plays a role in the regulation of angiogenesis in the colorectal region [39] and activates the Wnt/β-catenin pathway [40]. *HOXA13,* whose over-expression in UL has previously been described [21,34], is also associated with tumor size, microvascular invasion, angiogenesis, Wnt and TGFβ3 pathway in cancer [41,42,43]. *COL24A1,* a member of the collagen gene family, is related with vascular invasion and is proposed as a target for UL treatment [44]. Its overexpression in hepatocellular carcinoma leads to tumor and vascular invasion [45]. According to our study, it plays an intermediate role between ECM and angiogenesis in UL, being associated with a higher presence of H3K27ac in its promotor. *IGFL3* is implicated in TFGβ signaling in breast cancer [46], but was not previously linked to UL until this study. Epigenetic regulation of *IGFL3* in UL through H3K27ac could lead to dysregulation of TGFβ3 pathway in this tumor. Inhibition of any of these genes by directly targeting them or though histone acetylation/deacetylation treatment could lead to a decrease in cell proliferation, angiogenesis, ECM and other pathways involved in UL pathogenesis.

We also found hypoacetylation and downregulation of tumor suppressor genes such as *CD40, GIMAP8, IL15, GPX3* and *DPT*, with *CD40, GIMAP8 and GPX3* associated with UL for the first time in this study. *CD40* has antiangiogenic and pro-immune properties in other tumors [47,48]. We propose that *CD40* hypoacetylation and, therefore downregulation, would promote angiogenesis and hide tumor cells from the immune system. *GIMAP8* is *a* GTP-binding with a tumor suppressive role against breast cancer [49]. *IL15* contributes to excessive ECM production, tissue remodeling and leiomyoma growth [50]. It also controls migration, invasion, metabolism and angiogenesis, decreasing the number of blood vessels in prostate cancer [51]. Its hyperacetylation and consequent downregulation would contribute to an increment on UL vasculature. *GPX3* is a tumor suppressor that prevents migration and invasion through the Wnt pathway in gastric cancer [52,53]. *DPT* inhibits cell proliferation, interacts with decorin for TGF-β binding and plays an important role in cell–matrix interactions and matrix assembly [54]. Its hypoacetylation and downregulation would disrupt the process of collagen fibrils and activate the TGF-β signaling pathway. The recovery of gene expression of these tumor-suppressor genes could stop the development of UL. All in all, the dysregulation of these genes confirms that key processes of UL development are under histone acetylation control.

The interplay of H3K27ac-gene expression and cell signaling pathways can broaden the understanding of UL development and requires more attention. Hence, functional enrichment analysis of 82 DEGs regulated by H3K27ac was performed. This analysis revealed biological processes significantly deregulated in human UL that were mainly related to cell proliferation, cell signaling and cell transport and angiogenesis processes. Uterine leiomyoma is characterized by an uncontrolled proliferation, which is also a main feature of tumors [4,55,56]. In addition, cell communication and cell signaling is altered in tumors, contributing to the aberrant response to extracellular signals and enhancing tumor development that is characteristic of this kind of disease [56,57,58]. Initiation of tumor angiogenesis is one hallmark of cancer and a requirement for tumor progression [56]. Malignant cells require oxygen and nutrients to survive and proliferate, needing proximity to blood vessels to access the blood circulation system. The aberrant vascularization found in UL [59] can be triggered by a change in histone marks such as H3K27ac. Different growth factors and vascular genes mediate the angiogenic process, which as demonstrated in this study is regulated by epigenetic states of genes. Accordingly, hyperacetylation/upregulation of oncogenes related with angiogenesis and vascular invasion (*COL24A1, NDP* and *HOXA13)* and hypoacetylation/downregulation of angiogenesis-tumor suppressor genes (*CD40* and *IL15)* was identified in this study. In addition, we found cellular components significantly enriched in UL, which were mainly related to an alteration of extracellular matrix formation. Excessive synthesis and deposition of ECM deposition exerts a major role in the growth and stiffness of UL, contributing to clinical symptoms, such as abnormal bleeding and abdominal pain [1,60,61]. For this reason, ECM has been considered as a crucial target for UL therapeutics [61]. Herein, we found hyperacetylation/upregulation of ECM-associated oncogenes, such as *COL24A1* and *IGFL3,* and hypoacetylation/downregulation of ECM-associated tumor suppressor genes such as *IL15* and *DPT.*

To corroborate that histone acetylation H3K27 is really affecting the expression of tumor suppressor genes in UL, we inhibited HDACs, enzymes who catalyse histone deacetylation, in vitro by SAHA in HULP cells. Inhibiting HDACs upregulated the expression of hypoacetylated and downregulated tumor suppressor genes (*CD40, GIMAP8, IL15, GPX3 and DPT*) in HULP cells in vitro. HDACs inhibitors are widely used as anticancer drugs to treat many tumors in which histone acetylation is altered, increasing the accumulation of acetylated core histones. As a consequence, SAHA blocks cell proliferation and tumor growth in tumors such as hepatoid adenocarcinoma [62], myeloid leukemia [63] and prostate cancer [64]. The impaired histone acetylation in UL shown in this study opens insights into the role of these treatments as therapeutic options to treat this disease, as it does in other tumors. We have previously demonstrated that SAHA treatment inhibits cell proliferation, cell cycle, ECM, and TGF-β3 signaling in HULP cells, suggesting that histone deacetylation may be useful to treat UL [29]. Herein, we reinforce this hypothesis by proving that reversal of histone acetylation by SAHA in HULP cells upregulated hypoacetylated/downregulated tumor suppressor genes. These results together give importance to histone acetylation as a therapeutic approach for UL patients.

Based on these findings, dysregulated pathways involved in UL pathogenesis, such as cell proliferation, cell signaling and cell transport, angiogenesis or ECM formation, could be targeted for future therapeutics through histone acetylation reversion. This study provides insight into the role of histone acetylation in UL development. Further studies focused on new treatments targeting these histone modifications will be necessary to define an effective treatment of UL without side effects.

## 5. Conclusions

In this study, we found hyperacetylation/upregulation of oncogenes (*NDP, HOXA13, COL24A1 and IGFL3*) and hypoacetylation/downregulation of tumor suppressor genes (*CD40, GIMAP8, IL15, GPX3 and DPT*) in UL, which are related to the immune system, angiogenesis, invasion, altered metabolism, deposit of extracellular matrix, TGFβ3 and Wnt/β-catenin pathway dysregulation. In conclusion, gene regulation by H3K27 acetylation is involved in uterine leiomyoma pathogenesis through processes such as cell proliferation, cell signaling and cell transport, angiogenesis, ECM, Wnt and TGFβ pathway, and reversal of this acetylation could offer a therapeutic option for patients with uterine leiomyomas.

## Data Availability

The datasets analysed during the current study are available in the GEO repository, https://www.ncbi.nlm.nih.gov/geo/ (accessed on 10 September 2021).

## Supplementary Materials

The following are available online at https://www.mdpi.com/article/10.3390/biomedicines10061279/s1, Table S1. Differential expressed genes with peaks within the ±2 kb region from the TSS of the DEGs with FDR-adjusted *p*-value < 0.01 after limma analysis.
